# Supplementary material for: Toward a more informative representation of the fetal–neonatal brain connectome using variational autoencoder
Source: eLife. 2023 May 15;12:e80878. doi: 10.7554/eLife.80878 (PMC10241511; doi:10.7554/eLife.80878)
Supplement: Supplementary file 1. — (a) Preprocessing steps for the DBI and dHCP datasets. (b) Age prediction performance in separate age groups of DBI dataset using different latent representations. [file elife-80878-supp1.docx]

| **Preprocessing step** | **DBI** | **dHCP** |
| --- | --- | --- |
| Slice time correction | O | O |
| Discarding the first few slices | O | X* |
| De-spiking | O | X |
| Bias-field correction | O | O |
| Intensity scaling | O | X |
| Spatial smoothing | X | X |
| Data censoring | O** | X |
| Bandpass filtering | O** | O*** |
| Nuisance regression | O**^,^ **** | O^†^ |
| ICA-FIX | X | O |
| Detrending | X | O |
| Voxel-wise normalization | O | O |
| * : Not mentioned, ** : Simultaneously applied,  *** : Not included in dHCP preprocessing routine,  **** : Only with motion parameters,  ^†^ : Motion parameters + ICA-FIX noise components. | | |

**Supplementary File 1a.** Preprocessing steps for the DBI and dHCP datasets.

**Supplementary File 1b.** Age prediction performance in separate age groups of DBI dataset using different latent representations.

| **Representation  Method** | **Neonate (37-47 weeks)** | | **Fetus (19-39 weeks)** | |
| --- | --- | --- | --- | --- |
|  | MAE | vs. VAE | MAE | vs. VAE |
| VAE | 1.30$\pm$0.02 |  | 2.89$\pm$0.02 |  |
| Cortical Parcel | 1.36$\pm$0.01 | *p*<10^-6^ | 2.73$\pm$0.03 | *p*<10^-6^ |
| IC50 | 1.45$\pm$0.03 | *p*<10^-6^ | 3.26$\pm$0.08 | *p*<10^-6^ |
| IC100 | 1.48$\pm$0.03 | *p*<10^-6^ | 3.01$\pm$0.05 | *p*<10^-6^ |
| IC200 | 1.37$\pm$0.02 | *p*<10^-6^ | 3.03$\pm$0.04 | *p*<10^-6^ |
| IC300 | 1.33$\pm$0.02 | *p*<10^-6^ | 3.06$\pm$0.04 | *p*<10^-6^ |
| Melodic ICA | 1.32$\pm$0.02 | *p*<10^-6^ | 2.98$\pm$0.03 | *p*<10^-6^ |
